# Supplementary material for: Probiotic Strains Isolated from an Olympic Woman’s Weightlifting Gold Medalist Increase Weight Loss and Exercise Performance in a Mouse Model
Source: Nutrients. 2022 Mar 17;14(6):1270. doi: 10.3390/nu14061270 (PMC8950690; doi:10.3390/nu14061270)
Supplement: Supplementary file 1 [file nutrients-14-01270-s001.zip › nutrients-1617471-supplementary.pdf]

Supplementary data:

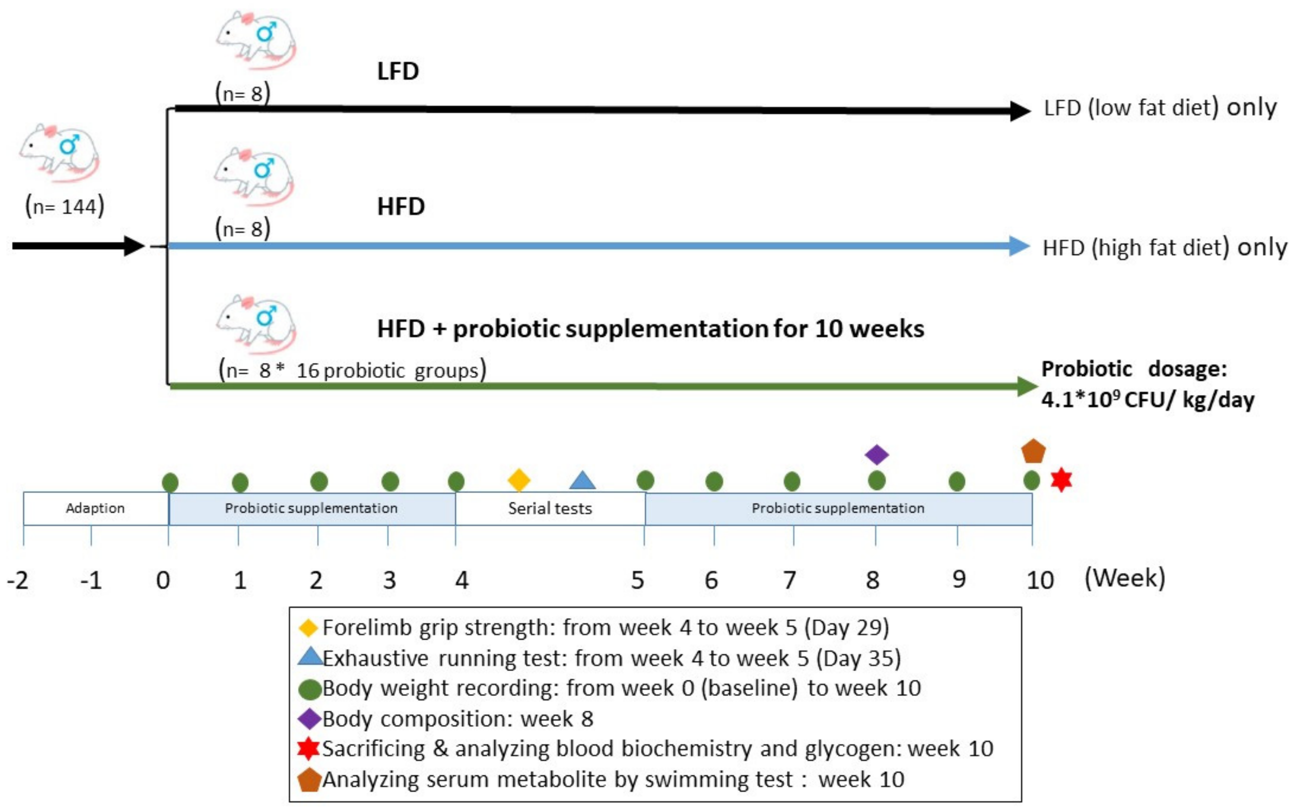

Figure S1. Experimental flowchart

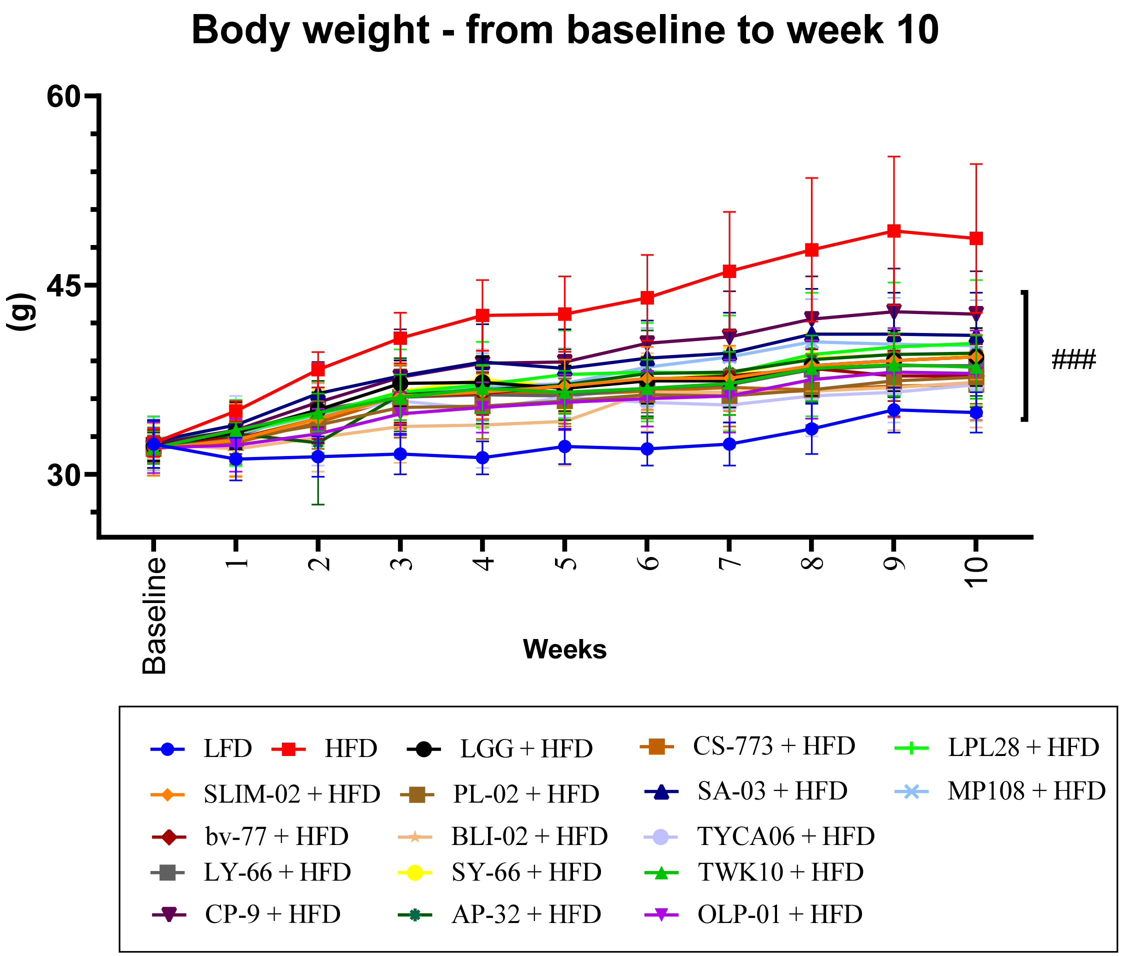

Figure S2. The Body weight change from week 0 to week 10

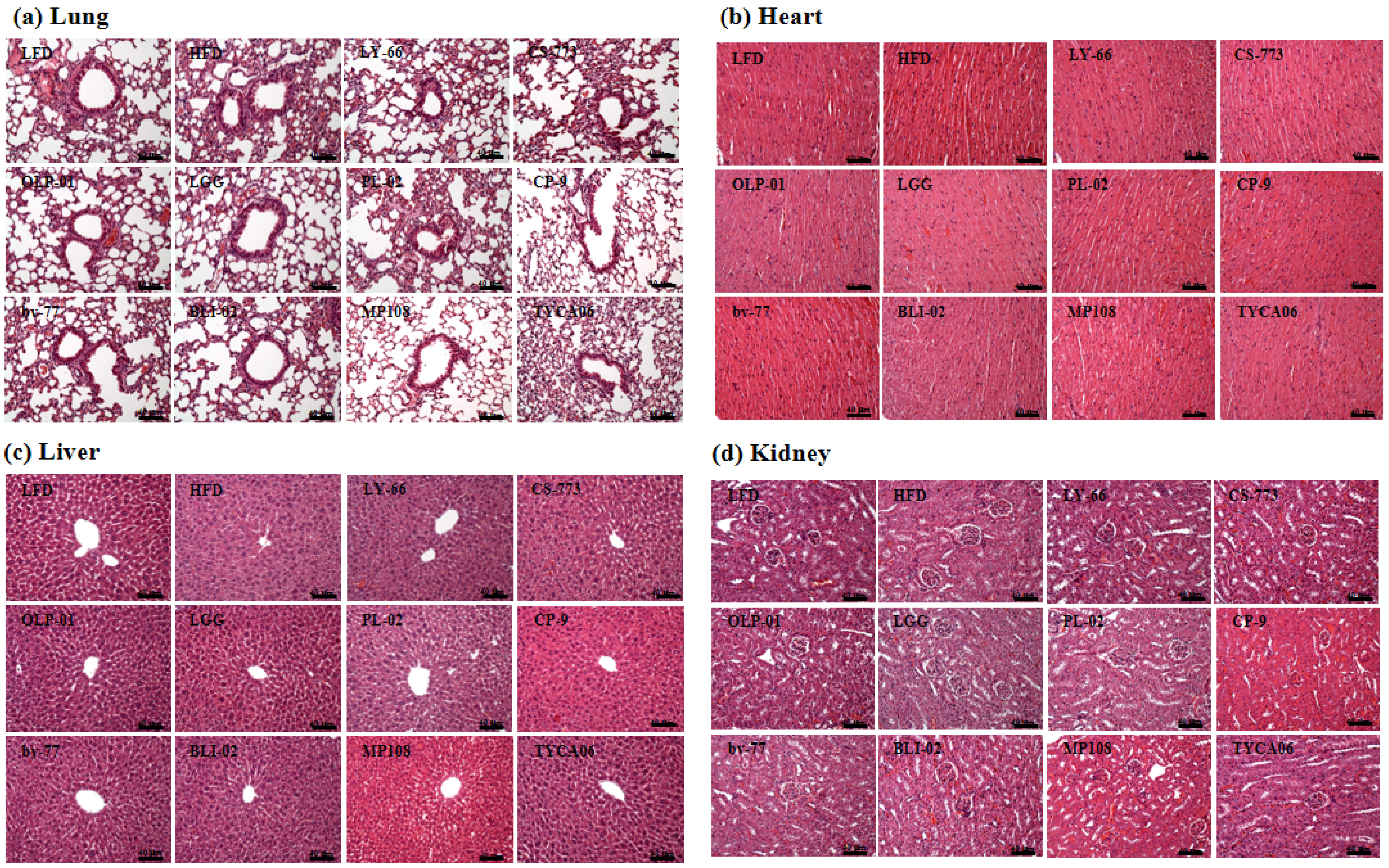

Figure S3. Histological sections of lung (a.), heart (b.), liver (c.) and kidney (d.) after feeding probiotics supplements for 10 weeks
